# Systemic evaluation of the risk of reoperations in pediatric populations with exotropia and esotropia: nested case–control study

**DOI:** 10.1038/s41598-023-39547-y

**Published:** 2023-08-01

**Authors:** Ju-Yeun Lee, Seong Jae Kim, Hyuna Kim

**Affiliations:** 1 Department of Ophthalmology, Myongji Hospital, Hanyang University College of Medicine, Goyang, South Korea; 2 Department of Ophthalmology, Gyeongsang National University School of Medicine and Gyeongsang National University Hospital, Jinju, Republic of Korea; 3 Institute of Medical Sciences, Gyeongsang National University School of Medicine, Jinju, Republic of Korea; 4 Biomedical Research Institute, Gyeongsang National University Hospital, Jinju, Republic of Korea; 5 Department of Ophthalmology, Soonchunhyang University Hospital, #59 Daesakwan-ro, Seoul, 04401 South Korea

**Keywords:** Risk factors, Epidemiology, Paediatric research

## Abstract

The study aimed to assess the risk of reoperations for strabismus in the pediatric population and to identify high-risk groups. This was a nationwide population-based study that used data from the Korean National Health Claims Database from 2008 to 2020. Two major cohorts were established based on initial strabismus: age-, sex-, and recruitment year-matched controls were randomly selected. Patients aged ≤ 9 years who underwent initial strabismus surgery for exotropia and esotropia were included, resulting in a total of 24,816 patients included to this study. The cumulative incidence of reoperations was 843 per 10,000 persons for exotropia, 1559 per 10,000 persons for esotropia. To assess the significant exposure factors, conditional logistic regression was performed to obtain odds ratio (OR) in each cohort. In patients with exotropia, the OR of reoperations was 4.26 times higher when the initial surgery is performed at younger age (earlier than 3-year-old) and 6.49 times higher when only one eye underwent than two eye. Similarly, in patients with esotropia, younger age (6.57 times) and unilateral surgery (7.20 times) were identified as common factors that increase the risk of reoperations. Based on the findings, special attention is recommended for patients younger than 3 years, especially those performed unilateral surgery as initial intervention in practical settings.

## Introduction

Strabismus is a common disorder that requires surgical correction to prevent the development of visual complications in the pediatric population. With advances in medicine and early screening systems, pediatric patients with strabismus requiring surgery have been diagnosed earlier than in the past. Consequently, the majority of first strabismus surgeries are performed in pediatric patients. Recurrent or residual strabismus is not rare after strabismus surgery. The reported proportion ranged from 5 to 59%, which is highly variable^1–7^. For both physicians and patients, it is difficult to determine the second surgery for strabismus, and several of them may encounter frustrating problems. There are well-established studies reporting risk factors for the recurrence of strabismus; however, few studies have assessed the risk factors for reoperations in pediatric patients with strabismus. In addition, most studies are regional and limited to a few centers; thus, it is necessary to evaluate the possibility of reoperations from a more comprehensive point of view.

Based on our previous studies, we accessed the use of the National Health Insurance Review and Assessment (HIRA) database containing all medical records of the entire Korean population. Since all medical information at the national level can be obtained from the HIRA data, reliable analysis of disease trends is possible in the entire pediatric population. To achieve a comparable level of evidence with the full cohort approach during the limited duration, we used a nested case–control study to analyze our nationwide data. In the nested case–control study, cases of a disease that occurred in a defined cohort were identified, and, for each, a specified number of matched controls was selected from among those in the cohort who did not develop the disease by the time of disease occurrence in the case. For several research questions, the nested case–control design potentially offers a reduction in costs and efforts of data collection and analysis compared with the full cohort approach, with a relatively minor loss in statistical efficiency. Using this study design, we aimed to investigate the risk of exposure to reoperations for strabismus in the entire pediatric population and elucidate high-risk groups based on systemic evaluation.

## Methods

This nationwide study was approved by the Institutional Review Board of Soonchunhyang University College of Medicine (IRB No. 2020-11-034) and adhered to the tenets of the Declaration of Helsinki. The requirement for informed consent was waived due to the retrospective design of the study and the anonymization of the data, which is approved by the Institutional Review Board of Soonchunhyang University College of Medicine.

### Data sources

We accessed health claims from 2008 to 2020 that were recorded via the HIRA service of South Korea. This claim database holds all health care information from both inpatients and outpatients using codes from the *Korean Standard Classification of Diseases* (KCD)*,* seventh revision, with a few changes related specifically to the Korean situation based on the tenth revision of the *International Classification of Diseases*. This database has been widely used to identify novel determinants of other diseases, and detailed information about the HIRA database has been presented and verified in our previous studies^8^.

### Participants and sample selection

All pediatric patients who were 9 years or younger who were identified as having had strabismus surgery (registration code S5173-6) during the 13-year study and had been continuously included in a medical plan for 6 months or longer were included in our estimates. All the participants with these codes were confirmed and registered by an ophthalmologist. Since the maximum age covered by health insurance for strabismus surgery is usually below 10 years of age for the South Korean insurance system, we only included patients aged ≤ 9 years to ensure accurate estimation. Based on the simultaneous diagnosis at the time of initial surgery, all patients were classified into exotropia (H5010-4, H5018-9, H5032-3) and esotropia (H500, H5002, H5008-9, H5030) groups. To help ensure that complex conditions were not included, except for initial exotropia or esotropia, all patients who had any record of paralytic strabismus or external ophthalmoplegia (H490-4, H498-9); disorders of binocular movement including conjugate gaze palsy, internuclear ophthalmoplegia, ocular motor apraxia (H510, H512, H518-9), Brown syndrome, Duane’s syndrome; or other mechanical strabismus (P5060, P5068-9, P5080) on the same day or before the index date of strabismus surgery were excluded. Most enrollees in this database were Asian. For all patients included in this study, age, sex, type of insurance, records of medical visits, date of diagnosis and treatment, and comorbidities from the HIRA database were used.

### Analyses

#### Establishing dataset and ascertainment of study groups

Participants who underwent two or more strabismus surgeries were included in the study group for each type of strabismus: initial exotropia or esotropia groups. Control group was defined as those who underwent only a single strabismus surgery during the study period. Participants who underwent reoperations in the initial exotropia group were matched with those who underwent only a single surgery (control group). Participants with esotropia were matched in the same manner. The participants in the control group were sorted using a random number generator. The matched controls were presumed to have been included concurrently with each reoperation participant. Therefore, after excluding a prior history of reoperations, we randomly selected four controls for each study patient by extracting age-, sex-, and index year-matched control groups from the eligible participants to prevent selection bias. We analyzed the medical claims data of possible exposure factors before the index date in the study and control groups.

#### Definition of exposure factors

Possible exposure factors for reoperations, including demographic, surgical, and ocular factors and perinatal or neurologic diseases were set. We defined the factors associated with the risk of reoperations as follows: (1) age, sex (2) income (high income/lower income supporting by medical care): Income was reflected by the type of copayment induction in the medical care system according to the gross income. It was used as a proxy for socioeconomic status, and medical care refers to lower income (3) retinopathy of prematurity (ROP) (H351), (4) problematic birth affected by maternal diseases (P000-8, P040-9), (5) problems during birth (P010-9, P020-9, P030-9), (6) respiratory disorders including hypoxia, respiratory distress syndrome, or apnea in newborns (P200-9, P210-9, P220-9, P240-9, P282-9), (7) intracranial hemorrhage (P100-9, P110-9, P120, P520-9), (8) other brain disorders including hydrocephalus, ischemia, convulsion, or coma (P910-9, P90), (9) endocrinologic disorders such as thyroid problems (P720-9), (10) attention deficit hyperactivity disorder (ADHD) (F900, F988), (11) laterality of surgery (unilateral, bilateral), (12) type of surgery (simple, complex, simple + complex) (for a simple surgery, only the rectus muscle was operated on; complex surgeries involved cases of operation of oblique muscles or cases of transposition, adjustable suture, or posterior fixations), (13) the number of operated muscles in one eye. These factors were collected based on the diagnostic codes confirmed by medical doctors in the HIRA system and used for conditional logistic regression analysis.

#### Statistics

Data handling and statistical analyses were performed by an independent data analyst (J. L.) specially trained by the HIRA institute for HIRA big data (2017). To compare the survival distribution, a unstratified log-rank test was used to compare the incidence of reoperations in the whole exotropia and the whole esotropia groups during the study period. To take full advantage of the matched data, conditional logistic regression was used to compute a crude odds ratio (OR) and adjusted OR for the study groups compared with the control groups. Adjustment for multiple statistical comparisons was performed, then p value < 0.0045 was considered to be significant. A 95% confidence interval was used for this analysis, and all results were presented as mean ± standard deviation (SD). SAS Enterprise Guide version 6.1 software program (SAS Inc., Cary, NC, USA) was used for all the analyses.

## Results

### Demographics

**Figure 1:**
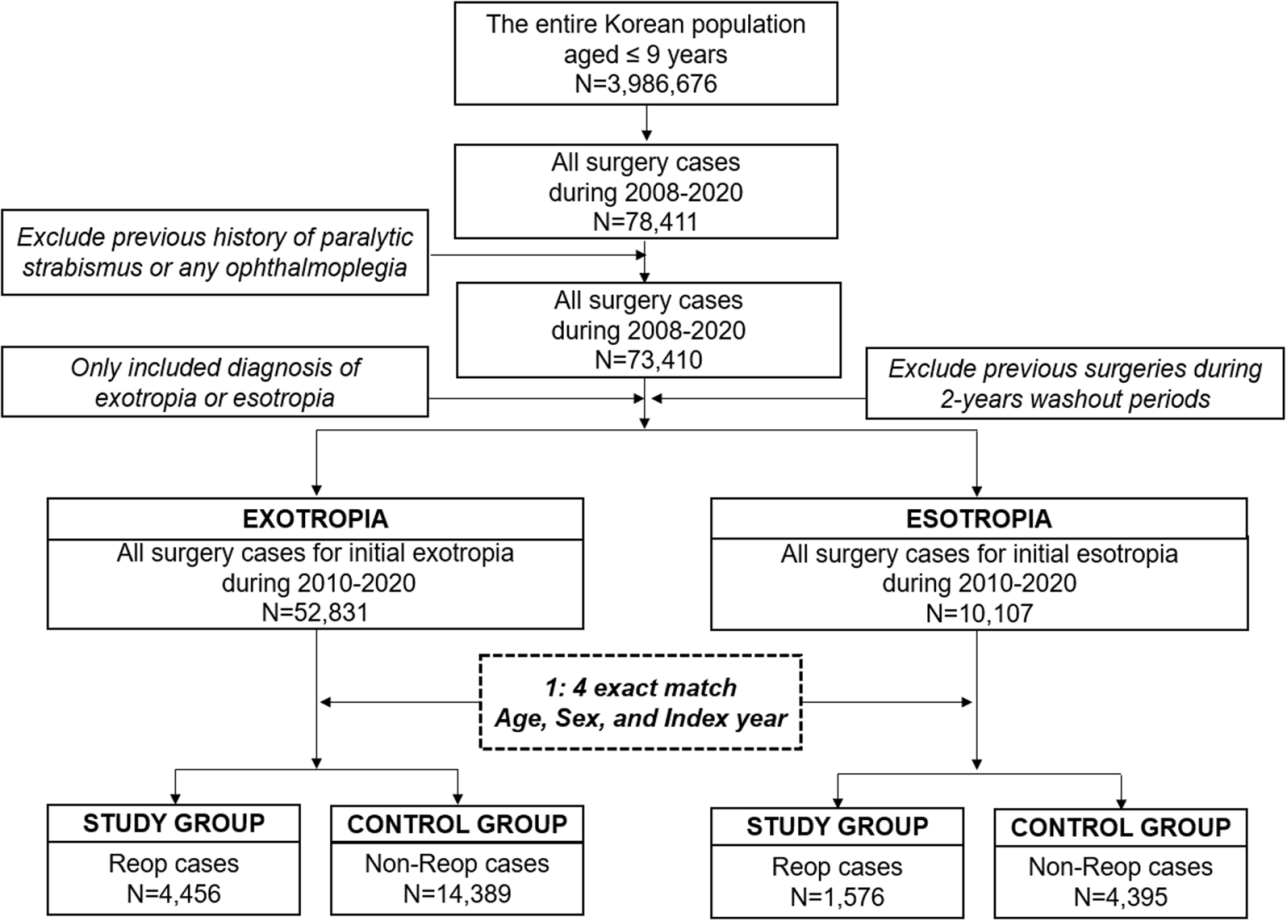
Identifying the study participants and controls in South Korea using nested case–control design. Among 78,411 participants who had undergone primary strabismus surgery, 18,845 participants are finally included in the exotropia cohort and 5971 participants in the esotropia cohort from 2010 to 2020.

A total of 78,411 participants underwent primary strabismus surgery during the enrollment period (2008–2020). After considering the wash-out periods, the incidental surgery cases from 2010 to 2020 were 62,938, including 52,831 initial exotropia cases and 10,107 cases of initial esotropia. The detailed identification process and demographics are shown in Fig. 1 and Table 1, respectively.

**Table 1:** Demographic of study and control groups in patients with two types of major strabismus (exotropia/exotropia) using national representative data (2008–2020) in South Korea.

Variable	Study group	Control group	*P* value
**Exotropia**	N = 4456	N = 14,389	
Age at the first op (years, SD)	4.7(1.7)	5.9(2.1)	< 0.001
Age at the second op (years, SD)	7.4(1.7)	–	
Males:females	1950:2506	6173:8216	0.315
Income^1^			0.296
Higher income	4348	14,094	
Lower income supporting by medical care	108	295	
Operation type			< 0.001
Simple^2^	3737	12,417	
Complex^3^	566	1647	
Simple + complex	153	325	
Operation laterality			< 0.001
Unilateral	3406	5395	
Bilateral	1050	8994	
Retinopathy of prematurity	143	414	0.265
Intracranial hemorrhage^4^	34	137	0.278
Other brain problems^5^	52	164	0.872
Problematic birth			
During delivery	26	138	0.016
By maternal medical condition^6^	15	43	0.646
Respiratory disorders^7^	119	454	0.110
Endocrinologic disorders^8^	21	59	0.598
**Esotropia**	N = 1576	N = 4395	
Age at the first op (years, SD)	3.0(2.1)	4.2(2.6)	< 0.001
Age at the second op (years, SD)	5.9(2.1)	–	
Males:females	726:850	2061:2334	0.576
Income^1^			0.344
Higher income	1534	4249	
Lower income supporting by medical care	42	146	
Operation type			< 0.001
Simple^2^	1260	3694	
Complex^3^	262	611	
Simple + complex	54	90	
Operation laterality			< 0.001
Unilateral	1020	1053	
Bilateral	556	3342	
Retinopathy of prematurity	156	363	0.048
Intracranial hemorrhage^4^	55	151	0.936
Other brain problems^5^	81	210	0.586
Problematic birth			
During delivery	32	111	0.292
By maternal medical condition^6^	5	30	0.124
Respiratory disorders^7^	136	413	0.388
Endocrinologic disorders^8^	12	61	0.052

*N* number; *SD* standard deviation.

^1^Income was reflected by the type of copayment induction in medical care system according to the gross income. It was used as a proxy for socioeconomic status. The type of insurance is divided into ordinary and medical care.

^2^Simple type means that only the rectus muscle was operated.

^3^Complex type means that cases of oblique muscle which was operated together, or cases of transposition, adjustable suture, or posterior fixations.

^4^It refers traumatic/nontraumatic intracranial hemorrhage.

^5^Brain problems include seizure, cerebral ischemia, convulsion, hydrocephalus.

^6^Maternal conditions include maternal infection, renal, circulatory, respiratory, hypertension, medication, chemotherapy, smoking or alcohol.

^7^Respiratory disorders include acute respiratory distress syndrome, apnea, neonatal aspiration syndrome, or asphyxia.

^8^Endocrinologic disorders include neonatal/congenital goiter, thyrotoxicosis, or thyroid function disorders.

### Incidence and trend of reoperations

**Figure 2:**
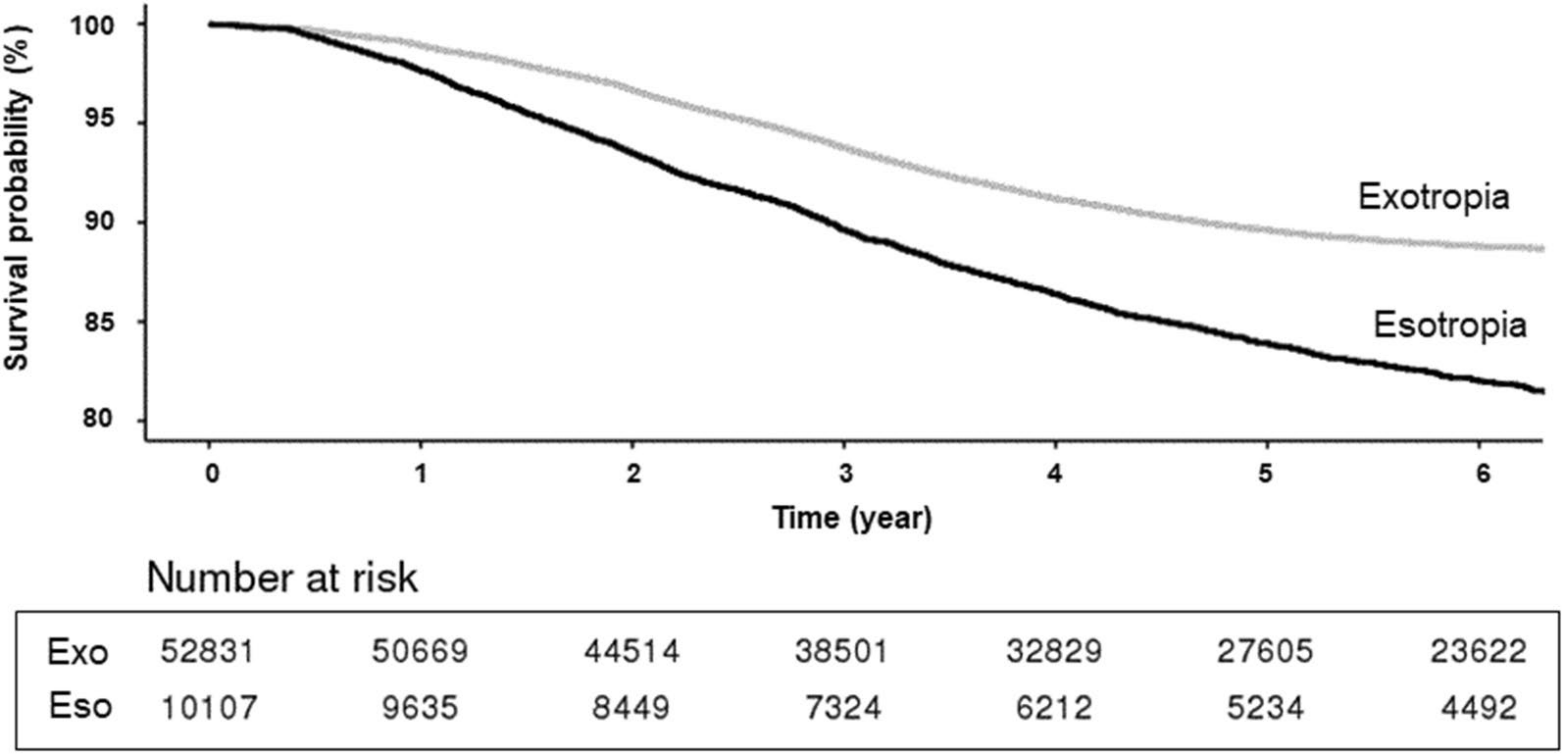
The survival probability of not undergoing another strabismus surgery in exotropia and esotropia cohort.

During the 10-year study, the cumulative incidence of reoperations was 843 per 10,000 patients in the initial exotropia group and 1559 per 10,000 patients in the initial esotropia group. The survival probability of not undergoing another strabismus surgery in each group during the study period is presented in Fig. 2 (log-rank, *p* < 0.01) In the exotropic patients, the interval between the first and second surgeries was 2.6 ± 1.5 years. In the esotropic patients, the interval between the first and second surgeries was 2.8 ± 1.8 years.

### Risk of reoperations based on established data of the initial exotropia group

**Figure 3:**
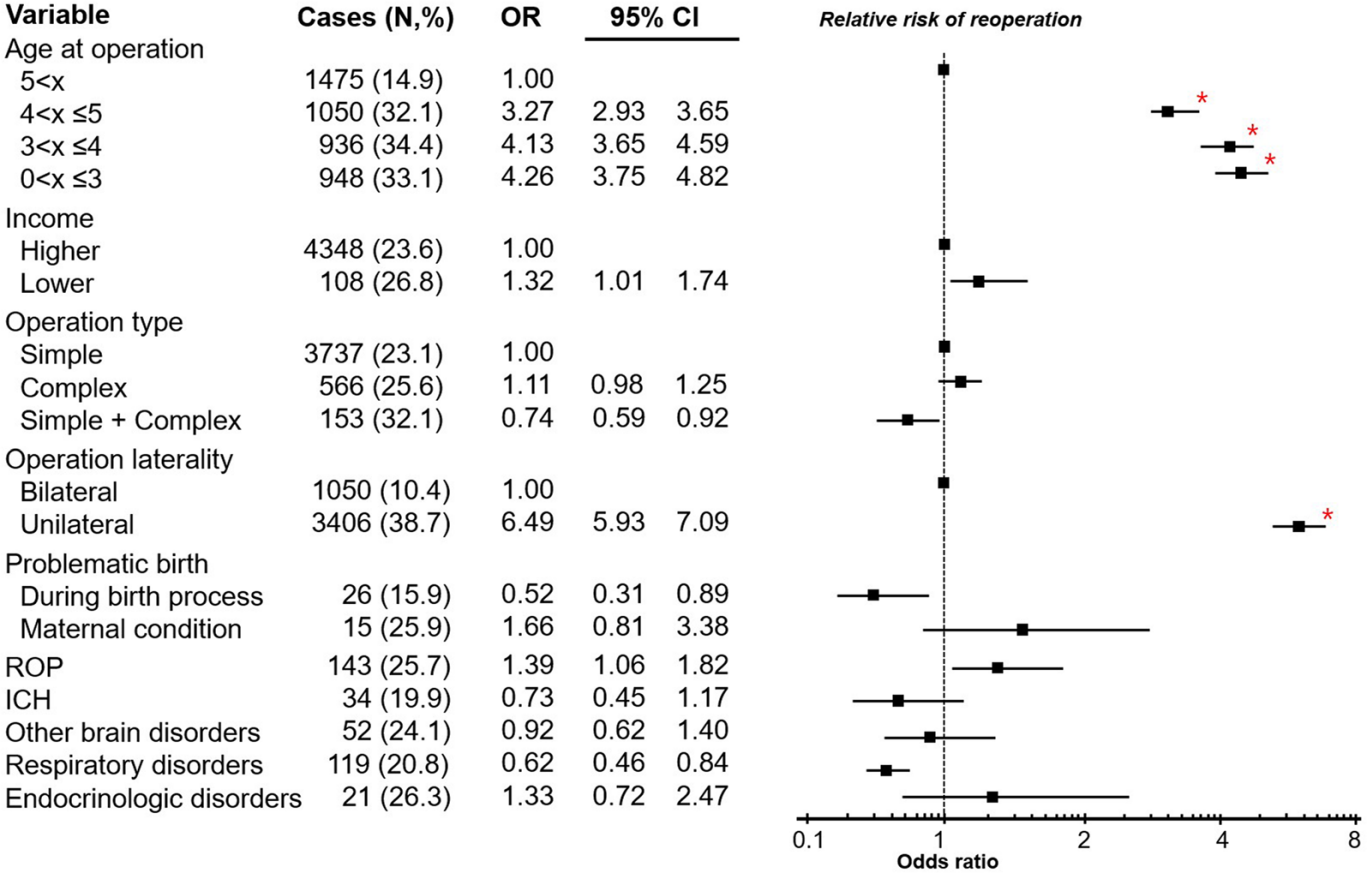
Risk of reoperations in the initial exotropia group using conditional logistic regression analysis. The early age at initial surgery, unilateral surgery are significant risks for reoperations. Error bands represents as 95% confidence intervals with relative risk of reoperation. *Statistically significant values.

In the established exotropia cohort, 4456 participants were finally included in the study group having reoperation, while 14,389 age-, sex-, and index year-matched participants were included in the control group not having reoperation. The conditional logistic regression analysis showed that the OR of reoperations was 4.26-fold higher (95% CI 3.75–4.82) for the 0–3 years, 4.13-fold higher (95% CI 3.65–4.59) for the 3–4 years and 3.27-fold higher (95% CI 2.93–3.65) for the 4–5 years age group than for the 5 years and older age group. The OR was 1.32-fold higher (95% CI 1.01–1.74) for the lower-income group and 1.39-fold higher (95% CI 1.06–1.82) for the participants with ROP, and they are not statistically significant after multiple statistical comparisons. The OR for the unilateral surgery group was 6.49-fold higher (95% CI 5.93–7.09) than the bilateral surgery group (p < 0.001) (Fig. 3).

### Risk of reoperations based on established data of the initial esotropia group

**Figure 4:**
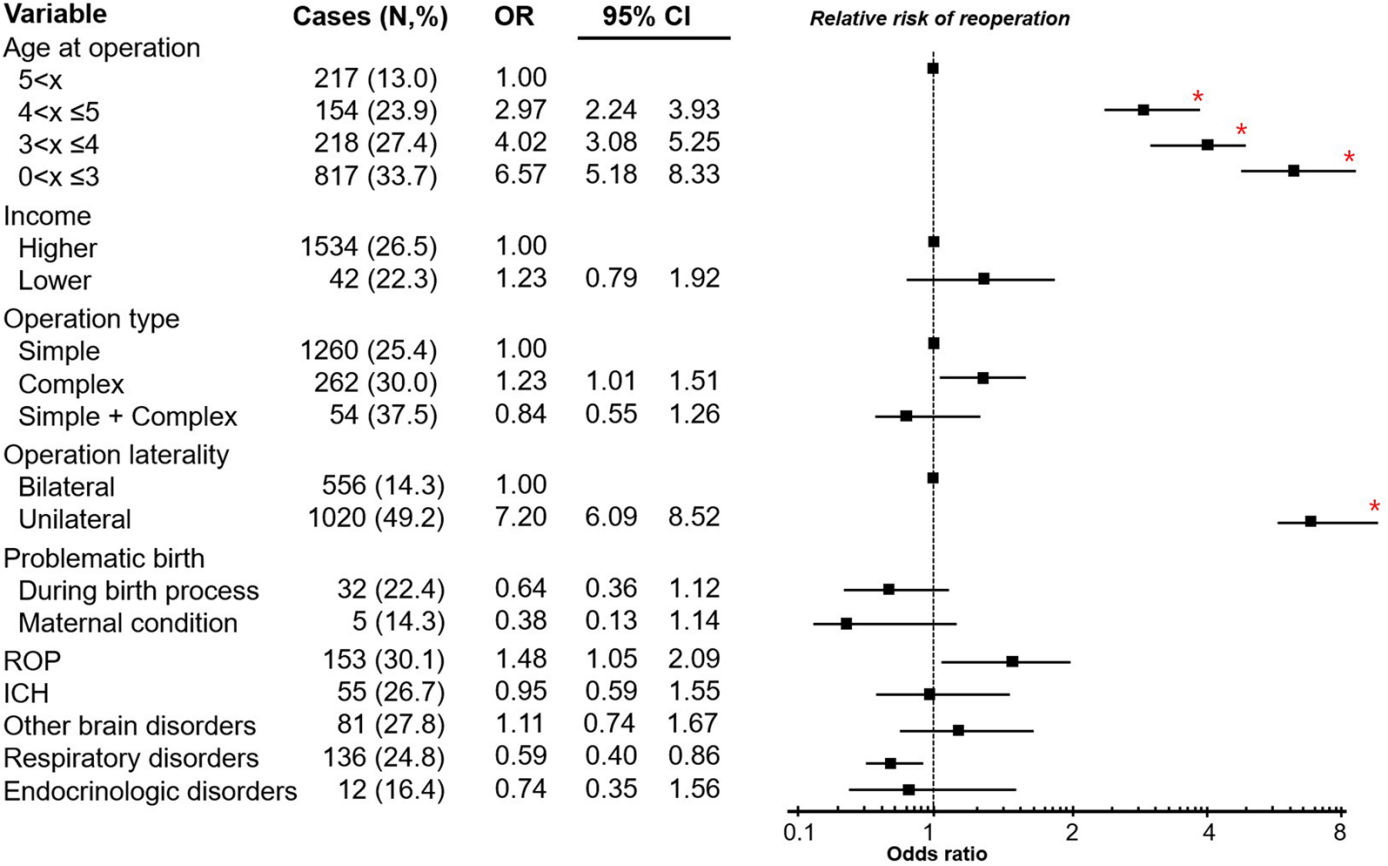
Risk of reoperations in the initial esotropia group using conditional logistic regression analysis. The early age at initial surgery, unilateral surgery are significant risks for reoperations. Error bands represents as 95% confidence intervals with relative risk of reoperation. *Statistically significant values.

In the established cohort for esotropia, 1576 participants were finally included in the study group having reoperation, while 4395 age-, sex-, and index year-matched participants were included in the control group not having reoperation. The conditional logistic regression analysis showed that the OR of reoperations was 6.57-fold (95% CI 5.18–8.33) higher for the 0–3 years age group, 4.02-fold (95% CI 3.08–5.25) higher for the 3–4 years age group, and 2.97-fold (95% CI 2.24–3.93) higher for the 4–5 years age group than for the 5 years and older age group. The OR for participants who underwent complex surgery was 1.23-fold higher (95% CI 1.01–1.51) than those who underwent simple surgery, and it was 1.48-fold higher (95% CI 1.05–2.09) for patients with ROP than for those without ROP. These differences are not statistically significant after multiple statistical comparisons. The OR for the unilateral surgery group was 7.20-fold (95% CI 6.09–8.52) higher than the bilateral surgery group (p < 0.001) (Fig. 4).

## Discussion

During the 10-year study period, we estimated the risk of reoperation for strabismus and assessed the possible risk factors. The cumulative incidence of reoperations was much higher in the initial esotropia group (1559 per 10,000 patients) than in the exotropia group (843 per 10,000 patients). In both cohorts, the common prognostic factors for reoperations were young age, unilateral surgery. Other perinatal systemic conditions, including endocrinological, neurologic, and respiratory disorders, were not significantly different for the major strabismus groups.

Several previous studies have investigated the risk factors of residual or recurrent strabismus^5–7,9,10^. Well-known risk factors include large preoperative deviation, distance/near disparity, age, stereopsis, and amblyopia in participants with initial exotropia, age at surgery, preoperative angle of deviation, hyperopic refractive error, or nystagmus in participants with initial esotropia. In another study, greater age at surgery, greater initial eso-deviation, and lateral incomitance were considered risk factors^11,12^. However, the definition of under- or over-correction was variable among researchers, even though the incidence and proportion of non-satisfying results of the first surgery were highly variable. Moreover, most previous studies have considered only ocular factors. As the occurrence and progression of strabismus may be closely related to developmental factors, systemic factors need to be evaluated together.

In clinical practice, it is meaningful to evaluate the possibility of another surgery, which may be a more direct issue for patients rather than the presence of strabismus. Based on several previous studies, decisions of reoperation or surgical factors were relatively variable depending on the medical center or surgeons in a nation, making it difficult to obtain representative nationwide results of reoperation. Moreover, there is a high possibility of follow-up loss in a single center during long-term studies, and it is challenging to collect a patient’s medical history in detail. To overcome these limitations, data need to be sufficiently large to represent general trends, and all relevant medical history should be obtained for a long time, even if patients change their hospitals or see other doctors. Therefore, we focused on a clear definition of ‘reoperations,’ which is recognized by medical records, established a nationwide cohort with long follow-up periods, collected all information on past medical histories, and statistically assessed significant prognostic factors for reoperations based on systemic evaluation in the entire population.

A few studies have reported reoperation for strabismus. Benson et al. reported the reoperation rate after the initial strabismus surgery as 15.7% in a single center for 21 years^2^. For cases of adult patients, Mills and the American Academy of Ophthalmology reported unplanned reoperation rates of up to 21% for comitant strabismus and up to 50% for thyroid ophthalmopathy^13^. Using the IRIS registry, Repka et al. reported a 6.72% reoperation rate one year after the initial surgery^3^. In the recent report from France, the 2-year reoperation rate was 7.7% in a cross-sectional analysis of medical claim data for the entire country^4^. The mean interval for the second surgery was 2.3 years, which was consistent with our findings of 2.63 years for the exotropia group and 2.76 years for the esotropia group. In the current study, the reoperation rate after the initial strabismus surgery was approximately 8% for exotropia and 15% for esotropia during the 10-year follow-up, which can be reliable and representative values for the entire pediatric population in South Korea.

Age has been demonstrated to be a powerful factor for reoperations that reflect poor fusion and early onset of strabismus.

Lastly, we observed a higher likelihood of reoperation in cases where unilateral surgery was performed than bilateral surgery. In both cohorts, among the study group who underwent reoperation, unilateral surgery accounted for the majority of cases (64–76%). Since the laterality of surgery is generally based on the amount of deviation, eye dominance, and the existence of unilateral amblyopia, which cannot be analyzed here, we could not define a precise surgical procedure based on laterality alone. In many cases, unilateral single-muscle surgery may be performed with a small deviation angle, resulting in a higher likelihood of the need for reoperation with unsatisfactory measurement. The effectiveness of two muscle surgery (bilateral lateral rectus (BLR) recession and unilateral recess-resect (RR) surgery) on the exotropia treatment varies among the studies depending on the type of exotropia or the study periods. Jeong and Hwang indicated that RR performed on the dominant eye yields better results than BLR^14^, and there are articles showed that augmented BLR with increased surgical amount outperforms RR^15,16^. A prospective study conducted by the Pediatric Eye Disease Investigator Group (PEDIG) over a three-year period, RR showed better results than BLR, it did not demonstrate statistical significance^17^. Therefore, it was concluded that it is not possible to recommend a specific surgical method. Some studies demonstrated that bilateral surgery is probably better for improving eye coordination than unilateral surgery^18^, or it has similar effects on motor alignment, stereoacuity, and quality of life^19–21^.

Further investigation is needed to verify our findings. Taken together, early unilateral surgery may lead to reoperation for strabismus.

This study has several limitations. First, we could not determine whether the second surgery was a reoperation for under-correction or over-correction. Additionally, we could not determine the subtypes of exotropia and esotropia. However, we focused on reoperations (number of surgeries), which can be extracted with high reliability, and combined the diagnosis and surgical type to increase accuracy. Second, insurance for surgery is usually limited to patients younger than 10 years of age. However, reoperations in South Korea are actively performed before the age of 10 years due to social insurance coverage, and a nested case–control design was selected for a retrospective view while increasing the level of evidence to compensate for this issue. Further long-term studies are required to confirm these findings. Finally, we were unable to obtain information regarding ethnicity. A recent HIRA publication reported that approximately 1% of the total population covered by health insurance comprises foreign nations. We assumed that most of the participants included in the study were Asian, although further analysis is needed to verify this assumption. In Korea, the frequency of exotropia is much higher than that of esotropia because the number of patients included in this study was five times greater than that of patients with esotropia. Therefore, there may be differences in the frequency of reoperations and risk factors between regions with a higher frequency of esotropia, such as North America and Europe. However, there were no significant differences in meaningful risk factors between exotropia and esotropia. Although we did not analyze the clinical records, including refractive error and the amount of deviation, we demonstrated the effect of systemic factors rather than ocular factors alone on reoperations.

In conclusion, we identified various prognostic factors for strabismus surgery based on the comprehensive cohort. Other than ocular factors, such as refractive error, several factors need to be considered when assessing the risk of reoperations. Our findings suggest that education on the risk of reoperations would be helpful to patients with significant prognostic factors.

## Data Availability

The data that support the findings of this study are available from HIRA but restrictions apply to the availability of these data, which were used under license for the current study, and so are not publicly available. Summary data are however available from the corresponding author upon reasonable request and with permission of HIRA.

## Abbreviations

CI: Confidence interval
HIRA: Health insurance review and assessment
KCD: Korean standard classification of diseases
OR: Odds ratio
ROP: Retinopathy of prematurity
SD: Standard deviation
